# Retraction statement: Gut microbiota and COVID‐19: A systematic review

**DOI:** 10.1002/hsr2.1460

**Published:** 2023-07-25

## Abstract

The following article, published online on {First published: 27 January 2023} in Wiley Online Library {https://onlinelibrary.wiley.com/doi/full/10.1002/hsr2.1080} has been retracted by agreement between the journal's Editor‐in‐Chief, Esmaeil Mehraeen and John Wiley & Sons Ltd. The retraction has been agreed given the journal has received evidence confirming that the peer review process of this paper was manipulated. As a result, the conclusions reported in the article are not considered reliable.

## References

[hsr21460-bib-0001] SeyedAlinaghi SA , Afzalian A , Pashaei Z , et al. Gut microbiota and COVID‐19: a systematic review. Health Sci Rep. 2023;6:e1080. 10.1002/hsr2.1080 36721396 PMC9881458

